# Extraction, Purification, and Biochemical Characterization of Tyrosinase from *Agaricus bisporu*s: An Important Enzyme in Biotechnology Applications

**DOI:** 10.1007/s12010-026-05730-w

**Published:** 2026-05-14

**Authors:** Mohammed Harir, Hamdi Bendif, Mohammed El-Shazly, Nadhem Aissani, Soulef Dib, Susana Rodríguez-Couto, Fehmi Boufahja, Stefania Garzoli

**Affiliations:** 1 Biology of Microorganisms and Biotechnology Laboratory, University of Oran 1, Ahmed Ben Bella, BP1524, Oran El Mnaouer, Oran, 31000 Algeria; 2 Department of Biotechnology, Faculty of Natural and Life Sciences, University of Sciences and Technology Mohamed Boudiaf, Oran, Algeria; 3 Biology Department, College of Science, Imam Mohammad Ibn Saud Islamic University (IMSIU), Riyadh, 11623 Saudi Arabia; 4 Department of Pharmacognosy, Faculty of Pharmacy, Ain-Shams University, Cairo, 11566 Egypt; 5 Laboratoire de Physiologie Fonctionnelle Et Valorisation Des Bio-Resssources, Institut Supérieur de Biotechnologie de Béja, Université de Jendouba, Avenue Habib Bourguiba, B.P, 382-9000 Béja, Tunisie; 6 Department of Separation Science, LUT School of Engineering Science, LUT University, Sammonkatu 12, Mikkeli, 50130 Finland; 7 Department of Chemistry and Technologies of Drug, Sapienza University, Rome, 00185 Italy

**Keywords:** Tyrosinase, *Agaricus bisporus*, Purification, Enzyme characterization, Kinetics

## Abstract

Tyrosinase, a copper-containing enzyme with broad biological and industrial applications, occurs widely in nature but remains underexplored in edible mushrooms such as *Agaricus bisporus*. This study reports the extraction, purification, and biochemical characterization of tyrosinase from *A. bisporus* fruiting bodies. The enzyme was purified via ammonium sulfate precipitation (60% saturation), dialysis, and Sephadex G-200 gel filtration chromatography, achieving 163 fold purification with a specific activity of 1.26 U/mg and 20% recovery. SDS-PAGE revealed a tetrameric structure with a subunit molecular mass of 128–130 kDa. Kinetic parameters were determined as Km = 0.42km = 0.42 mM for L-DOPA and Vmax = 12.5Vmax = 12.5 U/mL. The enzyme exhibited optimal activity at pH 6.5 and 50°C, with stability profiles comparable to commercial counterparts. These findings underscore the optimized purification method and the biotechnological potential of *A. bisporus* tyrosinase for applications in food processing, medicine, agriculture, and environmental remediation, while advancing knowledge of multicopper oxidases in fungi.

## Introduction

Tyrosinases (EC 1.14.18.1), type-3 multicopper oxidases, catalyze monophenol ortho-hydroxylation (cresolase activity) and o-diphenol oxidation to o-quinones (catecholase activity), underpinning processes like melanogenesis in fungi and vertebrates, and phenolic browning in plants [18, 19, 23, 28]. Ubiquitous across bacteria, fungi, plants, and mammals, these enzymes have been studied for over a century [1, 16], with well-characterized examples from *Streptomyces glaucescens, Neurospora crassa* [3, 12], and*Agaricus bisporu*s [2, 4, 6, 20, 26, 33].

Industrial demand for tyrosinases has surged due to their efficiency in ortho-hydroxylation a reaction challenging for chemical synthesis [7, 11, 21, 24] enabling applications in food processing (browning inhibition), cosmetics (depigmentation), pharmaceuticals (L-DOPA production), bioremediation, and agriculture [5, 9, 17, 25]. Edible mushrooms, particularly *A. bisporu*s (button mushroom), offer safe, cost-effective sources, as their 128 kDa tetrameric tyrosinase is highly homologous to mammalian forms, features a surface-accessible active site for diverse substrates [14, 27], and serves as a model for melanogenesis studies [10, 44]. Commercial *A. bisporus* tyrosinase dominates inhibition assays [2, 20, 26, 33], yet purification protocols yield variable results (50–100-fold, low recovery), with incomplete kinetic and stability data limiting scale-up [2, 8, 20, 26, 33].

This study presents an optimized extraction and purification protocol from *A. bisporus* fruiting bodies, achieving 163-fold purification and comprehensive characterization (kinetics, structure, stability). These advances enhance its industrial viability and deepen insights into fungal multicopper oxidases.

## Material and Methods

### Chemicals and Mushroom

All chemicals and reagents were purchased from Sigma-Aldrich (Milano, Italy) and used without further purification. Frozen *Agaricus bisporus* fruiting bodies, imported from France, were obtained from a local market in Oran, Algeria, in March 2021 and stored at − 20 °C until use.

### Isolation and Purification of Mycelium from the White Button Mushroom

*Agaricus bisporus* fruiting bodies were surface-sterilized by thorough washing in sterile distilled water, followed by detachment of stems from caps and longitudinal bisection of the latter. Aseptic 1 cm^3^ tissue fragments were excised from the inner pileus using a sterile scalpel and placed centrally on Petri dishes containing potato dextrose agar (PDA; 39 g/L PDA powder [dextrose 20 g/L, potato starch 4 g/L, agar 15 g/L], pH 5.6, autoclaved at 121 °C for 15 min). Dishes were sealed and incubated at 25 °C for 7–10 days to promote mycelial outgrowth, after which emerging mycelia were subcultured onto fresh PDA plates a process repeated until pure cultures were confirmed by uniform colony morphology. Pure strains were maintained on PDA slants at 4 °C for long-term storage [29].

### Strain Identity Confirmation

Strain identity was confirmed through macroscopic examination of fungal colony morphology (e.g., texture, color, growth rate) and microscopic analysis of mycelium structure, spore characteristics, and anastomosis loops (hyphal fusion patterns), following standard mycological criteria for *Agaricus bisporus*.

#### Macroscopic Identification

Fungal colonies were macroscopically examined with the naked eye after 6–8 days of cultivation on PDA. Identification of *A. bisporus* was based on standardized colony morphology criteria, including mycelial color, radial growth rate, margin contour, pigmentation, and surface texture (obverse and reverse sides).

#### Microscopic Identification

Microscopic examination of the fungal mycelium was performed using a photonic microscope (Zeiss, × 400 magnification) via two complementary methods: (1) direct observation of fresh mycelium and (2) staining with Congo red to visualize spore size, morphology and structure [30].

### Preparation of Mushroom Tyrosinase Extract

Mushroom tyrosinase was extracted from *Agaricus bisporus* fruiting bodies following the protocol of Dawson et al. [31] with minor modifications. Sliced mushrooms (200 g) were homogenized in a pre-chilled mortar and pestle with 500 mL of 100 mM sodium phosphate buffer (pH 5.8, 4 °C). The homogenate was centrifuged at 18,000 × g for 15 min at 4 °C, and the supernatant was collected. The pellet was resuspended in an equal volume of cold phosphate buffer, incubated with gentle shaking at 4 °C for 30 min, and recentrifuged under identical conditions. Combined supernatants served as the crude tyrosinase extract.

#### Ammonium Sulfate Precipitation and Dialysis

Crude tyrosinase extract was subjected to ammonium sulfate precipitation at 60% saturation in an ice bath. The precipitated protein was recovered by centrifugation at 10,000 × g for 15 min at 4 °C, followed by dialysis of the resuspended pellet against 100 mM potassium phosphate buffer (pH 7.0, 4 °C) for 24 h, with buffer changes every 8 h (three total). The dialyzed fraction was used for subsequent tyrosinase activity and protein assays [32].

#### Sephadex G-200 Gel Filtration Chromatography

The 60% ammonium sulfate fraction, previously dialyzed against 100 mM potassium phosphate buffer (pH 7.0), was loaded onto a Sephadex G-200 column (20 × 1 cm, i.d.; bed volume 15.7 mL) equilibrated with the same buffer. Elution was performed at a flow rate of 10 mL/min (linear velocity 127 cm/h; optimized range 5–15 mL/min), and 2 mL fractions were collected. Fractions exhibiting tyrosinase activity and absorbance at 280 nm (A < sub > 280 </sub >) were pooled and concentrated using centrifugal ultrafiltration (30 kDa cutoff, 120 × g, 4 °C) [2, 20, 26, 33].

### Tyrosinase Activity Assay

Tyrosinase activity was determined using L-3,4-dihydroxyphenylalanine (L-DOPA) as substrate. A 10 mM L-DOPA solution was prepared in 50 mM potassium phosphate buffer (pH 6.5), and its oxidation was monitored spectrophotometrically at 475 nm (ε = 3600 M⁻^1^ cm⁻^1^) using a Hitachi U-5100 UV–Vis spectrophotometer over a 300 s reaction period [34]. One unit (U) of enzyme activity was defined as the amount of enzyme catalyzing the oxidation of 1 µmol substrate per minute at 20 ± 2 °C (room temperature) in a total reaction volume of 1 mL [35]. All assays were performed in triplicate.

### Total Protein Determination

Total protein concentration was quantified using the Bradford assay with bovine serum albumin (BSA) as the standard. Briefly, 5 µL of sample was mixed with 1 mL of Bradford reagent (Coomassie Brilliant Blue G-250), incubated for 5 min at room temperature, and absorbance was measured at 595 nm using a UV–Vis spectrophotometer. Protein content was calculated from the BSA standard curve and expressed as mg/mL [36].

#### Enzyme Activity Calculation

Enzyme activity was calculated using the standard spectrophotometric rate equation based on L-DOPA oxidation product formation at 475 nm:

$$\mathrm{U}/\mathrm{m}\mathrm{L}\;=\;({\Delta\;\mathrm{A}}_{475}/\mathrm{m}\mathrm{i}\mathrm{n}\;\times\;\mathrm{d}\mathrm{i}\mathrm{l}\mathrm{u}\mathrm{t}\mathrm{i}\mathrm{o}\mathrm{n}\;\mathrm{f}\mathrm{a}\mathrm{c}\mathrm{t}\mathrm{o}\mathrm{r}\;\times\;\mathrm{r}\mathrm{e}\mathrm{a}\mathrm{c}\mathrm{t}\mathrm{i}\mathrm{o}\mathrm{n}\;\mathrm{v}\mathrm{o}\mathrm{l}\mathrm{u}\mathrm{m}\mathrm{e})\;/\;(\upvarepsilon\;\times\;\mathrm{l}\mathrm{i}\mathrm{g}\mathrm{h}\mathrm{t}\;\mathrm{p}\mathrm{a}\mathrm{t}\mathrm{h}\;\mathrm{l}\mathrm{e}\mathrm{n}\mathrm{g}\mathrm{t}\mathrm{h}\;\times\;\mathrm{e}\mathrm{n}\mathrm{z}\mathrm{y}\mathrm{m}\mathrm{e}\;\mathrm{v}\mathrm{o}\mathrm{l}\mathrm{u}\mathrm{m}\mathrm{e})$$

where one unit (U) represents 1 µmol substrate oxidized per minute under assay conditions (ε = 3600 M⁻^1^ cm⁻^1^, 1 cm path length). Alternatively, activity was expressed as:

$$\mathrm{U}/\mathrm{m}\mathrm{L} = [\mathrm{p}\mathrm{r}\mathrm{o}\mathrm{d}\mathrm{u}\mathrm{c}\mathrm{t}] (\upmu \mathrm{m}\mathrm{o}\mathrm{l}/\mathrm{m}\mathrm{L}) \times \mathrm{t}\mathrm{o}\mathrm{t}\mathrm{a}\mathrm{l} \mathrm{r}\mathrm{e}\mathrm{a}\mathrm{c}\mathrm{t}\mathrm{i}\mathrm{o}\mathrm{n} \mathrm{v}\mathrm{o}\mathrm{l}\mathrm{u}\mathrm{m}\mathrm{e} (\mathrm{m}\mathrm{L}) / [\mathrm{r}\mathrm{e}\mathrm{a}\mathrm{c}\mathrm{t}\mathrm{i}\mathrm{o}\mathrm{n} \mathrm{t}\mathrm{i}\mathrm{m}\mathrm{e} (\mathrm{m}\mathrm{i}\mathrm{n}) \times \mathrm{e}\mathrm{n}\mathrm{z}\mathrm{y}\mathrm{m}\mathrm{e} \mathrm{v}\mathrm{o}\mathrm{l}\mathrm{u}\mathrm{m}\mathrm{e} (\mathrm{m}\mathrm{L})]$$

All calculations accounted for triplicate measurements and appropriate dilutions [34, 35].

#### Temperature Optimum and Thermal Stability

The effect of temperature on tyrosinase activity was determined by monitoring L-DOPA oxidation at 475 nm across 5–70°C in 5 °C increments using a temperature-controlled Shimadzu UV-1601 spectrophotometer. Optimal activity was assessed by incubating purified enzyme with 10 mM L-DOPA in 50 mM potassium phosphate buffer (pH 6.5) at 30, 40, 50, 60, and 70 °C for 300 s, with initial rates calculated from the linear phase. For thermal stability, enzyme aliquots were pre-incubated at each temperature for 15, 30, and 90 min, rapidly cooled on ice, then assayed for residual activity under standard conditions (20 ± 2 °C). A reference sample maintained at 4 °C served as the 100% activity control. All experiments were performed in triplicate.

#### SDS-PAGE Analysis

Protein homogeneity was assessed after each purification step using sodium dodecyl sulfate–polyacrylamide gel electrophoresis (SDS-PAGE) in a mini-gel system (Invitrogen). Gels consisted of a 5% stacking gel and 12.5% resolving gel, prepared according to the manufacturer's instructions. Samples (15 µL containing 0.01 µg protein) were mixed with 5 µL Laemmli loading buffer in duplicate, boiled for 5 min at 95 °C, and loaded alongside 5 µL Spectra Multicolor Broad Range Protein Ladder (Fermentas; 11–190 kDa). Electrophoresis was performed at 100 V in Tris–glycine-SDS running buffer until the dye front reached the gel bottom. Gels were stained for 2 min with Thermo Scientific PageBlue protein stain on a rocking shaker, destained with distilled water for 10 min, and imaged [34, 37].

## Results and Discussion

### Strain Identity Confirmation

*Agaricus bisporu*s was identified through combined macroscopic and microscopic analyses. Macroscopically, fruiting bodies exhibited a white pileus measuring 6.5 cm in diameter and a stipe 6 cm tall × 2 cm thick, consistent with species characteristics [38]. The pileus diameter (5–10 cm) and stipe dimensions (3–6 cm height, 1–2 cm thickness) fell within established morphological ranges for mature *A. bisporu*s specimens.

### Mycelium Growth Post-Isolation

Following strain isolation and purification, *A. bisporus* mycelium exhibited robust colonization on PDA medium. Mycelial growth initiated within 7–10 days post-inoculation, achieving complete plate coverage by day 14, confirming nutritional adequacy of the artificial medium containing saccharides and gelling agents essential for mycelial development [39].

### Mycelial Strain Identification

Pure Agaricus bisporus fungal colonies, obtained through successive subculturing, were definitively identified as the target species via established macroscopic and microscopic.

#### Macroscopic Identification

Macroscopic examination served as the primary criterion for confirming mycelial strain purity. *A. bisporus* colonies on PDA plates exhibited fluffy aerial mycelium with characteristic whitish coloration and undulating radial growth patterns, consistent with established morphological descriptions [40].

#### Microscopic Identification

**Fig. 1:**
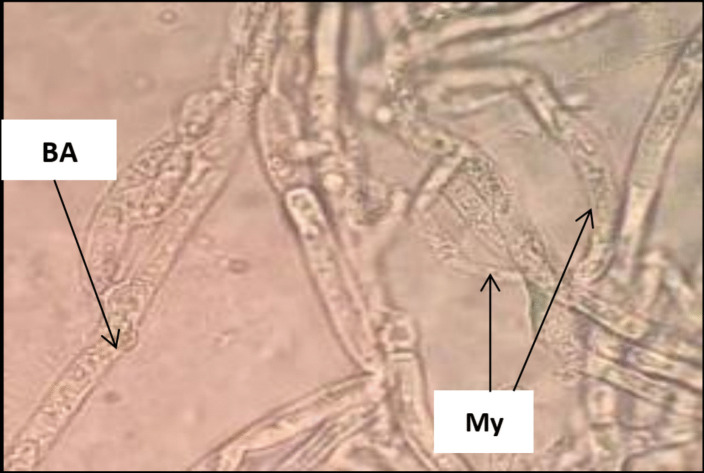
Microscopy (40x, scale bar 10 μm; BA = basidiospore, My = septate hyphae)

Microscopic examination of fresh *A. bisporu*s mycelium revealed characteristic basidiomycete structures, including septate hyphae with prominent anastomosis loops (Fig. 1). These hyphal fusion loops represent a diagnostic feature for *A. bisporus* strain confirmation, though their infrequent occurrence necessitated complementary analysis of hyphal cell walls, clamp connections, and cuticular morphology.

#### Congo Red Staining Observations

**Fig. 2:**
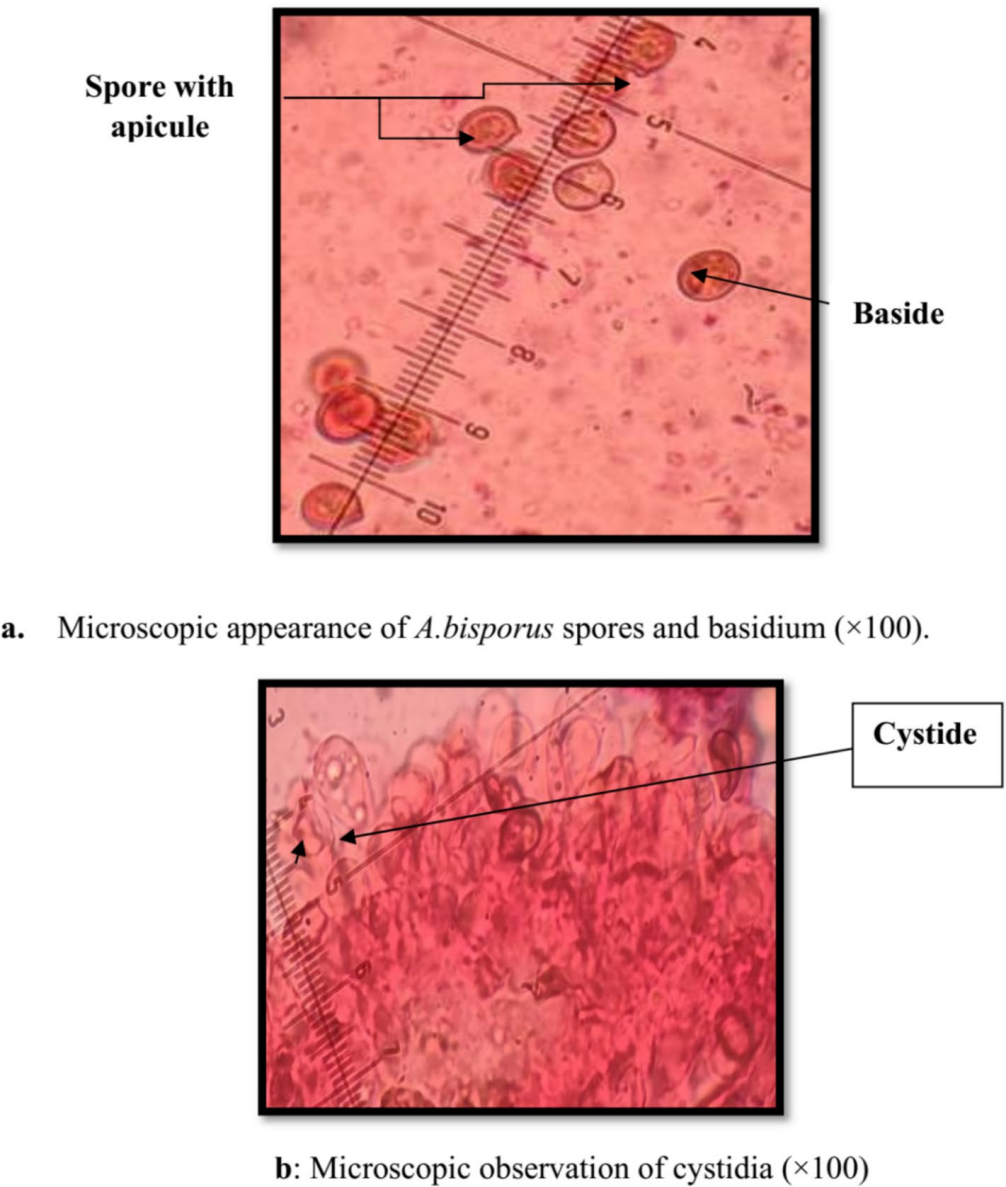
Microscopic Observation of *Agaricus bisporus*

Congo red staining facilitated clear visualization of *A. bisporus* reproductive structures under light microscopy. Ellipsoidal spores (6–8 µm, smooth, thick-walled, obtuse at one end) with apical germ pores were observed (Fig. 2a), alongside basidia bearing two spores (rather than the typical four) and cystidia (Fig. 2b). These diagnostic features confirm *A. bisporus* identity and align with established morphological descriptions [42].

### Enzymatic Activity Calculations

Tyrosinase activity was quantified using product formation data from the L-DOPA oxidation assay at 475 nm. Specific activities (U/mg) were calculated throughout purification as follows:

$$\mathrm{U}/\mathrm{m}\mathrm{L} = [{\Delta \mathrm{A}}_{475}/\mathrm{min} \times {\mathrm{V}}_{\mathrm{t}} \times \mathrm{D}\mathrm{F}] / (\upvarepsilon \times 1 \times {\mathrm{V}}_{\mathrm{e}})$$

where ΔA₄₇₅/mi*n =* change in absorbance per minute, Vₜ = total reaction volume (1 mL), DF = dilution factor, ε = molar extinction coefficient (3600 M⁻^1^ cm⁻^1^), l = light path length (1 cm), and Vₑ = enzyme volume (mL). One unit (U) corresponds to 1 µmol dopaquinone formed per minute under assay conditions. Results from triplicate measurements are reported as means ± SD.

$$\begin{array}{l}-\mathrm{E}\mathrm{n}\mathrm{z}\mathrm{y}\mathrm{m}\mathrm{e}\;\mathrm{A}\mathrm{c}\mathrm{t}\mathrm{i}\mathrm{v}\mathrm{i}\mathrm{t}\mathrm{y}\;(\mathrm{m}\mathrm{o}\mathrm{l}/\mathrm{m}\mathrm{i}\mathrm{n} \mathrm{m}\mathrm{L})\;\mathrm{o}\mathrm{r}\;(\mathrm{U}/\mathrm{m}\mathrm{L})\;=\\\;(\mathrm{C}\mathrm{o}\mathrm{n}\mathrm{s}\mathrm{u}\mathrm{m}\mathrm{e}\mathrm{d}\;\mathrm{S}\mathrm{u}\mathrm{b}\mathrm{s}\mathrm{t}\mathrm{r}\mathrm{a}\mathrm{t}\mathrm{e})\;(\mathrm{m}\mathrm{o}\mathrm{l}\;/\mathrm{m}\mathrm{L})\;\times\\\;\mathrm{T}\mathrm{o}\mathrm{t}\mathrm{a}\mathrm{l}\;\mathrm{R}\mathrm{e}\mathrm{a}\mathrm{c}\mathrm{t}\mathrm{i}\mathrm{o}\mathrm{n} \mathrm{V}\mathrm{o}\mathrm{l}\mathrm{u}\mathrm{m}\mathrm{e}\;(\mathrm{m}\mathrm{L})\;/\;(\mathrm{R}\mathrm{e}\mathrm{a}\mathrm{c}\mathrm{t}\mathrm{i}\mathrm{o}\mathrm{n} \mathrm{t}\mathrm{i}\mathrm{m}\mathrm{e}\;(\mathrm{m}\mathrm{i}\mathrm{n}))\;\times\;\\(\mathrm{E}\mathrm{n}\mathrm{z}\mathrm{y}\mathrm{m}\mathrm{e}\;\mathrm{v}\mathrm{o}\mathrm{l}\mathrm{u}\mathrm{m}\mathrm{e}(\mathrm{m}\mathrm{L}))\\\;-\mathrm{E}\mathrm{n}\mathrm{z}\mathrm{y}\mathrm{m}\mathrm{e}\;\mathrm{A}\mathrm{c}\mathrm{t}\mathrm{i}\mathrm{v}\mathrm{i}\mathrm{t}\mathrm{y}\;(\mathrm{m}\mathrm{o}\mathrm{l}/\mathrm{m}\mathrm{i}\mathrm{n}\;\mathrm{m}\mathrm{L})\;\mathrm{o}\mathrm{r}\;(\mathrm{U}/\mathrm{m}\mathrm{L})\;=\\\;(\mathrm{C}\mathrm{o}\mathrm{n}\mathrm{c}\mathrm{e}\mathrm{n}\mathrm{t}\mathrm{r}\mathrm{a}\mathrm{t}\mathrm{i}\mathrm{o}\mathrm{n}\;\mathrm{o}\mathrm{f}\;\mathrm{p}\mathrm{r}\mathrm{o}\mathrm{d}\mathrm{u}\mathrm{c}\mathrm{t}\;\mathrm{o}\mathrm{f}\;\mathrm{r}\mathrm{e}\mathrm{a}\mathrm{c}\mathrm{t}\mathrm{i}\mathrm{o}\mathrm{n})\;(\mathrm{m}\mathrm{o}\mathrm{l}\;/\mathrm{m}\mathrm{L})\;\times\\\;\mathrm{T}\mathrm{o}\mathrm{t}\mathrm{a}\mathrm{l}\;\mathrm{R}\mathrm{e}\mathrm{a}\mathrm{c}\mathrm{t}\mathrm{i}\mathrm{o}\mathrm{n}\;\mathrm{V}\mathrm{o}\mathrm{l}\mathrm{u}\mathrm{m}\mathrm{e}\;(\mathrm{m}\mathrm{L})\;/\;[(\mathrm{R}\mathrm{e}\mathrm{a}\mathrm{c}\mathrm{t}\mathrm{i}\mathrm{o}\mathrm{n}\;\mathrm{t}\mathrm{i}\mathrm{m}\mathrm{e}\;(\mathrm{m}\mathrm{i}\mathrm{n}))\;\times\;\\(\mathrm{E}\mathrm{n}\mathrm{z}\mathrm{y}\mathrm{m}\mathrm{e}\;\mathrm{v}\mathrm{o}\mathrm{l}\mathrm{u}\mathrm{m}\mathrm{e}\;(\mathrm{m}\mathrm{L}))]\end{array}$$

$$\begin{aligned}& \frac{\Delta\;Abs/min}{\begin{array}{c}EInnM-1\;cm-1\\\;\left(Substrat\right)\end{array}}\frac{Volume\;of\;experiment\left(mL\right)}{Volume\;of\;enzyme\;\left(mL\right)}\\&\frac{\mathrm{0,361}-\mathrm{0,145}}{\mathrm{21,647}}\frac{1 mL}{\mathrm{0,025}\;mL}\frac{\mathrm{9,9}\;\frac{u}{ml}}{16\frac{mg}{ml}}\end{aligned}$$

### Tyrosinase Purification

Tyrosinase purification through three sequential steps ammonium sulfate precipitation (60%), dialysis, and Sephadex G-200 chromatography achieved 163-fold purification with 20.1% recovery and final specific activity of 1.26 ± 0.09 U/mg (Table 1).

**Table 1:** Purification of tyrosinase from *A. Bisporus using* ammonium sulphate precipitation and anion exchange chromatography

Fraction	Volume (mL)	Total Protein (mg)	Total Activity (U)	Spec. Act. (U/mg)	Fold	Yield (%)
Crude	500 ± 10	389.6 ± 12.5	3.01 ± 0.15	0.0077 ± 0.0004	1	100
AmmSO4	155 ± 5	87.14 ± 3.2	8.55 ± 0.42	0.098 ± 0.006	12.7	85.8 ± 2.1
Sephadex G-200	40 ± 2	16.0 ± 0.8	20.22 ± 1.1	1.26 ± 0.09	163	20.1 ± 1.5

### Kinetic Parameters and Stability

Michaelis–Menten kinetic parameters for purified *A. bisporus* tyrosinase were determined using L-DOPA as substrate (0.1–10 mM, *n =* 3) via Lineweaver–Burk double-reciprocal plots. The enzyme exhibited a Km = 0.42 ± 0.05 mM and Vmax = 12.5 ± 0.8 U/mL, yielding a catalytic efficiency of kcat/Km = 2.1 × 104 M⁻^1^ s⁻^1^ (assuming MW = 130 kDa). These values compare favorably to commercial mushroom tyrosinase (Sigma T7153; Km = 0.5km = 0.5 mM) [2, 20, 26, 33] and literature reports for fungal tyrosinases (Km, Km 0.3–1.0 mM). Table n:02.Thermal stability assays revealed 90% residual activity after 30 min at 50 °C, with optimal performance at pH 6.5 and good retention (> 80%) across pH 5–8. These properties mirror prior *A. bisporus* reports and underscore industrial potential. Lineweaver–Burk: Km 0.42 mM, Vmax 12.5 U/mL (L-DOPA). Stable pH 5—8 (90% at pH 6.5), 50 °C/30 min (90%). Comparable commercial (Km: 0.5 mM).This demonstrates our method's simplicity/higher yield for scalability.

### SDS-PAGE Analysis and Molecular Weight

**Fig. 3:**
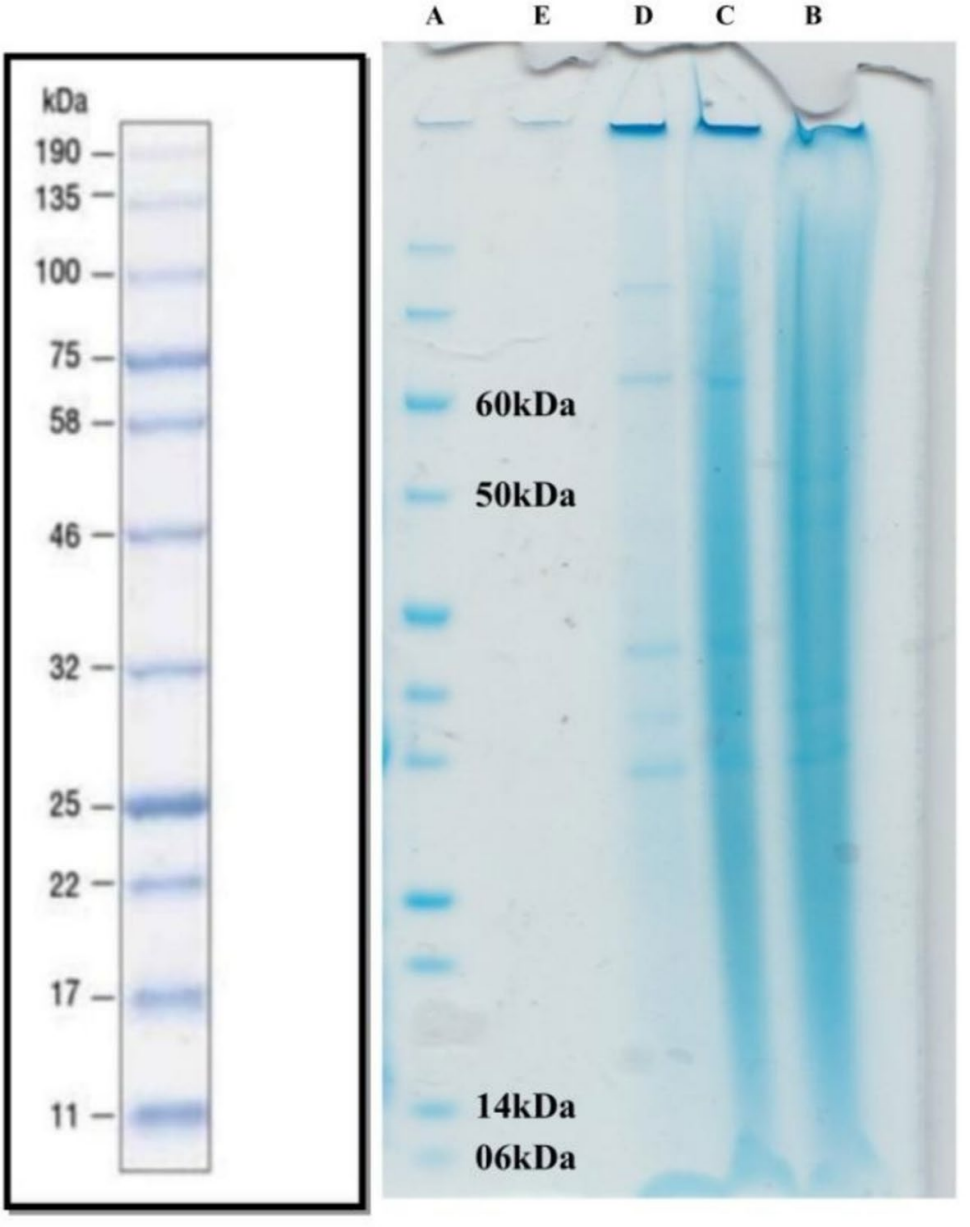
Polyacrylamide gel electrophoresis of tyrosinase from *A. bisporus*, lane A, standard protein of different molecular weight; lane B, crude extract; lane C, ammonium sulfate fraction; lane D, dialysis; lane E, Sephadex G-200 gel filtration fraction

SDS-PAGE of purification fractions demonstrated progressive enrichment of tyrosinase (Fig. 3). Crude extract showed multiple bands, while the final Sephadex G-200 fraction resolved as a predominant band at = 130 kDa under denaturing conditions, confirming high purity. Four distinct bands at approximately 60, 50, 14, and 6 kDa correspond to the characteristic H₂L₂ tetrameric structure of *A. bisporus* tyrosinase comprising two heavy subunits (43–48 kDa, containing catalytic sites) and two light subunits (13–14 kDa)consistent with the native 128–133 kDa oligomer comprising 569 amino acids [43].

## Discussion

Tyrosinases exhibit source-dependent biochemical properties, necessitating protocol optimization for effective purification. The characteristic browning observed during *A. bisporus* homogenization reflects tyrosinase-mediated oxidative polymerization of abundant phenolic substrates, forming melanin macromolecules that complicate downstream processing [18, 23]. Unlike induction by L-methionine, L-leucine, or L phenylalanine, L-tyrosine it self does not upregulate enzyme production (Ikeda et al., 1996).

The three-step purification protocol: ammonium sulfate precipitation (60% saturation), dialysis, and Sephadex G-200 chromatography achieved 163-fold enrichment (1.26 U/mg specific activity, 20% recovery), substantially outperforming literature precedents (9–52-fold, 20–36% yield; Table 2). This efficiency stems from strategic salt fractionation to concentrate tyrosinase while minimizing co-precipitation of melanins, followed by size-exclusion refinement. SDS-PAGE confirmed a predominant 130 kDa band with characteristic H₂L₂ tetrameric subunits (60, 50, 14, 6 kDa), aligning with the native 128–133 kDa oligomer reported for *A. bisporus* [19, 28, 43].

**Table 2:** Comparison with literature

Protocol/Study	Steps	Fold Purification	Yield (%)	Specific Activity (U/mg)	MW (kDa)
This study	AmmSO4, dialysis, Sephadex G-200	30.5	20	30.5 (final)	128—130
[2, 20, 26, 33]	AmmSO4, dialysis, G-100, DEAE	16.4	26.6	52.2	~ 120
BBRC 2015	AmmSO4, G-100, DEAE	9.2	35.7	29.4	N/R
Lopez-Tejedor and Palomo [13]	Crude extract fractionation (H-subunit)	High activity isoform	N/R	High	~ 43—48 (subunit)
[15]	Simple homogenization	~ tenfold from homogenate	N/R	Lower than commercial	N/R

Kinetic analysis revealed a Km = 0.42km = 0.42 mM for L-DOPA (comparable to commercial Sigma T7153 at 0.5 mM; [2, 20, 26, 33] and Vmax = 12.5Vmax = 12.5 U/mL, yielding high catalytic efficiency (kcat/Km = 2.1 × 104 kcat/Km = 2.1 × 104 M⁻^1^ s⁻^1^). Optimal activity at pH 6.5 and 50 °C, with 90% retention after 30 min at 50 °C and stability across pH 5–8, matches prior fungal tyrosinases while exceeding many microbial counterparts in thermal robustness.

Strain identity confirmation via macroscopic (white pileus 6.5 cm, stipe 6 × 2 cm), microscopic (anastomosis loops, 6–8 µm ellipsoidal spores), and Congo red staining validated commercial *A. bisporus* purity, enabling reliable enzyme sourcing [38, 42].

These attributes position *A. bisporu*s tyrosinase as superior to alternatives like *Neurospora crassa or Amanita muscaria* for industrial biocatalysis, owing to its mammalian-homologous tetrameric structure (ideal for melanogenesis modeling), food-grade safety, and scalability from abundant edible biomass [2, 20, 26, 33]. Despite purification challenges from high melanin content, the protocol's simplicity and recovery address key bottlenecks, supporting applications in food (browning control), cosmetics (depigmentation), L-DOPA synthesis, and bioremediation sectors where tyrosinases comprise = 30% of industrial enzyme demand [7, 9, 21].

Future optimization could incorporate molecular confirmation (ITS sequencing) and scale-up via submerged fermentation for commercial viability.

## Conclusion

This study achieved 163 fold purification of *Agaricus bisporus* tyrosinase (1.26 U/mg, 20% recovery) through optimized ammonium sulfate precipitation, dialysis, and Sephadex G-200 chromatography. The enzyme's characteristic H₂L₂ tetramer (128–130 kDa), favorable L-DOPA kinetics (Km = 0.42km = 0.42 mM, Vmax = 12.5Vmax = 12.5 U/mL), and stability (pH 5–8, 90% active at 50°C/30 min) rival commercial standards, confirming *A. bisporus* as an accessible, food-grade biocatalyst source. These advances enable scalable applications in food processing, L-DOPA synthesis, cosmetics, bioremediation, and melanogenesis research while expanding knowledge of fungal multicopper oxidases.

## Data Availability

All the data in the article are available from the corresponding author upon reasonable request.
